# Regional variations in test requiring patterns of general practitioners in Spain

**DOI:** 10.3109/03009734.2011.606927

**Published:** 2011-10-29

**Authors:** María Salinas, Maite López-Garrigós, Julián DÍaz, Mario Ortuño, Martin Yago, Begoña Laíz, Arturo Carratala, Virtudes Chinchilla, Goizane Marcaida, Enrique Rodriguez-Borja, Angel Esteban, Marcos Guaita, Cristina Aguado, Miguel A. Lorente, Emilio Flores, JoaquÍn Uris

**Affiliations:** ^1^Department of Clinical Laboratory, Hospital Universitario de San Juan, Alicante, Spain; ^2^Department of Clinical Laboratory, Hospital Francesc Borja, Gandía, Valencia, Spain; ^3^Department of Clinical Laboratory, Hospital de la Ribera, Alzira, Valencia, Spain; ^4^Department of Clinical Laboratory, Hospital de Requena, Valencia, Spain; ^5^Department of Clinical Laboratory, Hospital La Fe, Valencia, Spain; ^6^Department of Clinical Laboratory, Hospital Clinico Universitario, Valencia, Spain; ^7^Department of Clinical Laboratory, Hospital General Universitario de Alicante, Spain; ^8^Department of Clinical Laboratory, Hospital General Universitario de Valencia, Spain; ^9^Department of Information Systems, Roche Diagnostics, Valencia, Spain; ^10^Department of Public Health, University of Alicante, Spain

**Keywords:** Appropriateness, clinical chemistry, health care costs, laboratory test utilization, quality indicators

## Abstract

**Objective:**

To analyze the requesting patterns for a range of laboratory tests ordered in 2009 from eight laboratories providing services to eight health areas, using appropriate indicators.

**Design:**

Indicators measured every test request per 1,000 inhabitants, and indicators that measured the number of tests per related test requested by general practitioners were calculated. The savings generated, if each Health Care Department achieved the appropriate indicator standard, were also calculated. Laboratory Information System registers were collected, and indicators were calculated automatically in each laboratory using a data warehouse application.

**Results:**

There was a large difference in demand for tests by health areas. The ratio of related tests also showed a great variability. The savings generated if each Health Care Department had achieved the appropriate indicator standard were €172,116 for free thyroxine, €18,289 for aspartate aminotransferase, and €62,678 for urea.

**Conclusions:**

Considerable variability exists in general practitioners' demand for laboratory tests.

## Introduction

Laboratory expenses have increased considerably in recent years (1). The growing prominence of laboratory science in diagnostic practices (2) and information technology advances that enhance test accessibility (3) increases test use. However, the same factors also predispose to over-use and misuse.

The proportion of inappropriate test requests ranges from 4.5% to 95%, in studies of medical records (4) using implicit and explicit criteria. These studies are complex, difficult to conduct, and generally not cost-effective, because tests are usually a relatively low-cost item. Studies to compare ordering behavior are a less expensive way to obtain information useful for optimizing laboratory test use.

Demand for laboratory testing services by primary care in eight Health Care Departments of the Valencia Health Agency in 2009 was evaluated in this study using a range of pathology tests representing 90% of requests in general practice to compare primary care test ordering behavior.

## Material and methods

The study was carried out under the auspices of the Valencia Health Agency to compare 2009 tests requested by general practitioners (GPs). The network was created by issuing two surveys to 13 laboratories (March 2008). The inclusion criteria were a common laboratory information system (LIS) (Omega, Roche Diagnostic^®^, Spain) and a data warehouse application capable of stratifying LIS results among clients (in-patients, primary care patients, etc.). The eight laboratories included provide services to hospitalized, emergency, specialized, and primary care patients whose samples are obtained in primary health care centers for hematology and blood chemistry tests. The population served in each Health Care Department is shown in Table I. In stage 2, we defined and validated the registers and indicators and created a common database. In stage 3 (March 2010), the results were presented confidentially to participating hospitals at a meeting. Each laboratory representative knew only the identification of their own data. A 2-month period of reflection and suggestions followed.

**Table I.** Tests/1,000 inhabitants in 2009 in different health care areas. These areas received health services from different Health Care Departments to which the different laboratories participating in the study pertain.

	Health care areas (total population December 2009)							
	A (197,029)	B (254,233)	C (274,233)	D (271,218)	E (55,282)	F (372,138)	G (357,267)	H (233,075)
General								
Alanine aminotransferase	383	364	350	293	380	209	298	280
Alkaline phosphatase	85	149	54	117	73	75	113	98
Aspartate aminotransferase	169	224	140	107	95	202	298	280
Cholesterol	318	363	337	339	422	215	351	276
Creatinine	511	364	345	324	387	219	311	276
Gamma-glutamyltranspeptidase	156	341	314	106	376	193	249	267
Glucose	524	373	356	350	408	239	368	278
HDL cholesterol	228	239	200	250	306	199	329	207
Potassium	341	167	157	309	203	97	165	135
Total bilirubin	154	139	100	94	143	95	103	56
Triglycerides	317	361	333	338	423	199	348	275
Urea	381	112	91	145	117	212	285	56
Uric acid	313	340	329	173	363	183	293	265
Anemia profile								
Iron	102	207	86	89	100	52	185	73
Ferritin	92	146	87	87	118	45	121	110
Transferrin	38	15	59	12	78	10	34	19
Rheumatic profile								
C-reactive protein	167	147	54	58	115	110	71	75
Calcium	156	43	284	100	129	44	68	37
Phosphate	85	37	253	95	86	30	40	32
Rheumatoid factor	24	33	57	64	31	26	44	42
Thyroid profile								
Free thyroxine	95	110	42	18	68	117	74	42
Thyrotropin	152	140	160	82	226	117	147	100

The daily LIS registers (tests requested by GPs) were collected and indicators were calculated using a computer application based on data warehouse and OnLine Analytical Processing OLAP cubes (Omnium, Roche Diagnostic^®^, Spain). Every test request per 1,000 inhabitants (22 chemistry tests) and the appropriate indicators that measured the number of tests per related test requested (aspartate aminotransferase/alanne aminotransferase, urea/creatinine, free thyroxine/thyrotropin) by GPs were calculated.

The savings generated, if each Health Care Department achieved the appropriate indicator standard (0.2 for aspartate aminotransferase/alanine aminotransferase, 0.1 for urea/creatinine, and 0.25 for thyroxine/thyrotropin), were also calculated (5).

## Results

Table I shows the test requests per 1,000 inhabitants per Health Care Department in 2009 grouped according to the tests most used in primary care. There was a large difference in demand for tests by health areas.

The appropriate indicators (ratio of related tests) are shown in Table II. The ratio of related tests also showed a great variability.

**Table II.** Value of the appropriate indicators that measure the number of tests per related tests requested by General Practitioners in different Health Care Departments.

	Health care areas							
	A	B	C	D	E	F	G	H
Key Performance Indicator (KPI)								
Aspartate amino transferase/ Alanine amino transferase	0.435	0.611	0.404	0.372	0.246	0.962	1.000	1.000
Urea / Creatinine	0.725	0.285	0.283	0.455	0.298	0.968	0.918	0.198
Free thyroxine / Thyrotropin	0.587	0.701	0.269	0.255	0.306	1.000	0.504	0.419

The savings generated, if each Health Care Department had achieved the appropriate indicator standard, were €253,083: €18,289 for aspartate aminotransferase, €62,678 for urea, and €172,116 for thyroxine.

## Discussion

Studies to evaluate clinical laboratory activities are a tool increasingly used to compare performance between organizations using the same measures and the same measurement system. It took 2 years to set up the laboratory network and data collection process and to identify suitable indicators for comparing primary care requesting performance.

Primary care demand for tests is growing (6) in terms of requests per inhabitant or per doctor. Our results demonstrate the broad variability of demand for tests by primary care on a test-by-test basis. It is not easy to establish a relationship between variability and center characteristics. All departments participating in the study, except one, are in an urban and coastal zone and are also influenced by seasonal tourism. The Valencian Community ranks the fourth most populated autonomous community of Spain, in a very populated country. There is no significant difference between the percentages of population aged >65 years in different centers. Five hospitals are university hospitals, but it is not possible to establish a relationship between the indicator values and the type of hospital.

It is strange to observe the great differences in serum glucose requesting, and the duplicated demand for potassium and sodium (the sodium results were not reported because they were identical to the potassium in all Health Care Departments) from one health area to another, where the prevalence of hypertension and need for monitoring are probably similar. Probably, different uses of lipids and uric acid measurements in the prevention of hypercholesterolemia and metabolic syndrome, respectively, will be responsible for large differences in requests (7). Moreover, uric acid is requested out of habit. In addition, despite evidence to the contrary, most GPs believe that an annual physical examination detects subclinical illness (8). Many report using laboratory tests for screening purposes with little evidence to support the practice, although it has become less frequent (9) as evidence-based guidelines have come into use and medical cost containment has become a concern.

Larsson et al. (5) recommend using serum alanine aminotransferase and alkaline phosphatase as the only liver markers and demonstrated the enormous savings that would result from reducing demand for aspartate aminotransferase, bilirubin, and gamma-glutamyltranspeptidase, which varied widely in the study.

Laboratory tests to diagnose anemia are subject to both under-use and over-use (10). It is well known that iron alone should not be ordered as part of an anemia investigation. However, it is surprising that certain departments are requesting from 3 to 10 times more iron than transferrin tests. Despite the iron test being cheap, its uselessness as an isolated test and the high within-person biological variation of serum iron indicate that early establishment of corrective measures is required to reduce its isolated request and to highlight the need for iron and transferrin to be used in combination. It is an archaic habit, important to change soon.

The variability in requests for rheumatic and thyroid disease tests is also noteworthy; for instance, free thyroxin is requested despite evidence that it may be generated after abnormal thyrotropin in primary care patients. There is also a huge difference in the requesting patterns of calcium, possibly an ‘under-used’ test in some areas. Treatable primary hyperparathyroidism disease in patients who are generally free of somatic manifestations is most often diagnosed when routine biochemical testing shows an elevated serum calcium level. The advent of automated laboratory serum analyzers in the 1960s allowed for the detection of ‘asymptomatic hypercalcemia’, and the incidence of hyperparathyroidism increased 5-fold during the next two decades. However, nowadays hyperparthyroidism can be again overlooked due to the disappearance of analyzers of continuous type. Calcium is a parameter whose use should be encouraged.

Probably, high demand for C-reactive protein tests in area A indicated more frequent use to detect/monitor infectious/rheumatoid diseases, when compared to other areas.

In certain health areas, urea and creatinine requests were similar, and transferase requests were identical (1:1 demand ratio). However, there is evidence indicating that serum urea provides little more information than serum creatinine alone (11), as also applies to the two transferases. We only studied the economic effects of decreasing three serum chemistry test ratios, and the total savings would be great. If we use these figures to calculate the total savings for all health care areas in Valencian Community, and more tests ratios, it would represent an enormous saving.

The great differences observed in the study in all test requests in such a large population are inconsistent with the goal of providing equitable care and apparently reflect local traditions (5). The differences are probably due to individual variations in clinical practice; however, they still undermine laboratory cost-effectiveness efforts and could be changed (12), preferably through regional strategies developed by consensus between GPs and clinical pathologists and formalized in health care governance measures.

Given the large difference in requesting patterns between health areas, the first step in optimizing laboratory test use would be to establish basic reflex testing strategies. Implementation of these measures would then be monitored using appropriate indicators, which actually vary greatly from one district to another, after setting clear goals. Nowadays most tests are added by automated analysers in accordance with algorithms. Previous process mapping will be needed in less automated laboratories. Laboratory directors should be aware that any strategy implemented to reduce inappropriate test use will reduce the number of tests processed by personnel and will appear to diminish theoretical performance. However, higher-quality diagnostic information would be obtained. In addition, unexpected results and false positives would decrease, and, concomitantly, so would the number of diagnostic tests performed and specialist consultations required for their interpretation, which are an important source of anxiety for patients. This is where the contribution of laboratory professionals is critical, by providing fewer, more selective, optimally effective diagnostic data.

Laboratory data are involved in 70% of clinical decisions (13); however, laboratory costs usually account for a very small proportion of overall health care costs. Patients should receive the full benefit from laboratory medicine, and it is important to encourage the use of some tests, such as serum calcium.

This study revealed that considerable variability exists in the use of laboratory tests by eight health areas. It also identified appropriate indicators that can be applied across a spectrum of clinical laboratories, and which thus may be useful for examining requesting patterns. The study highlighted the need to unify demand by optimizing the use of appropriate tests through interdepartmental communication and rigorous application of scientific evidence (14).
